# Can vibrotactile stimulation and tDCS help inefficient BCI users?

**DOI:** 10.1186/s12984-023-01181-0

**Published:** 2023-05-04

**Authors:** Kyungho Won, Heegyu Kim, Daeun Gwon, Minkyu Ahn, Chang S. Nam, Sung Chan Jun

**Affiliations:** 1 School of Electrical Engineering and Computer Science, Gwangju Institute of Science and Technology, Gwangju, South Korea; 2 Department of Computer Science and Electrical Engineering, Handong Global University, Pohang, South Korea; 3 School of Computer Science and Electrical Engineering, Handong Global University, Pohang, South Korea; 4 Edward P. Fitts Department of Industrial and Systems Engineering, North Carolina State University, North Carolina, USA; 5 Artificial Intelligence Graduate School, Gwangju Institute of Science and Technology, Gwangju, South Korea

**Keywords:** BCI, BCI illiteracy, Performance variation, Vibrotactile stimulation, Brain stimulation

## Abstract

Brain-computer interface (BCI) has helped people by allowing them to control a computer or machine through brain activity without actual body movement. Despite this advantage, BCI cannot be used widely because some people cannot achieve controllable performance. To solve this problem, researchers have proposed stimulation methods to modulate relevant brain activity to improve BCI performance. However, multiple studies have reported mixed results following stimulation, and the comparative study of different stimulation modalities has been overlooked. Accordingly, this study was designed to compare vibrotactile stimulation and transcranial direct current stimulation’s (tDCS) effects on brain activity modulation and motor imagery BCI performance among inefficient BCI users. We recruited 44 subjects and divided them into sham, vibrotactile stimulation, and tDCS groups, and low performers were selected from each stimulation group. We found that the latter’s BCI performance in the vibrotactile stimulation group increased significantly by 9.13% (*p* < 0.01), and while the tDCS group subjects’ performance increased by 5.13%, it was not significant. In contrast, sham group subjects showed no increased performance. In addition to BCI performance, pre-stimulus alpha band power and the phase locking values (PLVs) averaged over sensory motor areas showed significant increases in low performers following stimulation in the vibrotactile stimulation and tDCS groups, while sham stimulation group subjects and high performers showed no significant stimulation effects across all groups. Our findings suggest that stimulation effects may differ depending upon BCI efficiency, and inefficient BCI users have greater plasticity than efficient BCI users.

## Background

Brain-computer interface (BCI) constitutes an interface between a computer and the human brain that allows people to control a computer using their brain activity without any body movements [1, 2]. BCI can be categorized according to its control features, in which motor imagery BCI is an active BCI that uses the brain signals generated when people imagine body movement, such as moving both hands, feet, and tongue [3, 4]. Motor imagery BCI applications have helped people by providing a game controlled by brain signals [5, 6] that improves rehabilitation training [7, 8]. Compared with other BCI control features, motor imagery is intuitive, so its BCI is natural to users, and it offers a greater sense of control than other BCI types because motor imagery BCI is active. However, challenging issues remain in BCI, including motor imagery BCI and other BCI systems. BCI researchers have reported that a significant proportion of subjects (approximately 15–30%), who are referred to as ‘BCI-illiterate’, failed to achieve controllable BCI performance [9–12]. Those subjects were unable to generate the distinct brain activity pattern during a BCI task, so a machine learning-based classifier failed to extract stable features even though the subjects performed the BCI task. Although BCI researchers have proposed many applications and feature extraction algorithms, BCI illiteracy remains a significant challenge.

To overcome BCI illiteracy so that everyone can achieve controllable BCI performance, researchers have approached the issue from multiple perspectives. One approach is to develop new feature extraction algorithms to identify hidden and robust features to improve BCI performance in the hope that they can extract control features from BCI-illiterate as well as good performers [13–16]. For example, Riemannian approaches calculate the Riemannian distance between the template covariance matrices and a single trial covariance matrix to determine the minimum distance class, which achieved improved performance [16], while deep learning techniques have achieved dramatically improved performance in subject-specific and cross-subject BCI models through multiple hidden layers [13, 14]. However, although those proposed advanced feature extraction algorithms improved BCI performance, it is unclear whether deep learning methods can improve everyone’s BCI performance. For example, Lee et al. [13] proposed subject-independent BCI using deep convolutional neural networks (CNNs), and tested it with 54 subjects. The subjects achieved better BCI performance compared to existing classification methods with respect to subject-specific and subject-independent BCI performance evaluation. However, the deep learning technique did not solve the BCI illiteracy issues fully, as low BCI performers still remained and achieved less than 50% accuracy (near chance level) in binary class motor imagery. Moreover, in Xu et al.’s [17] investigation with eight different EEG datasets, the mean BCI performance was lower in the datasets with many subjects than in those with fewer subjects. It can be inferred that datasets with many subjects may contain more who are BCI-illiterate, and whose performance did not improve, while high BCI performers who had achieved controllable performance already with existing algorithms improved their performance further.

Another approach extends beyond feature extraction algorithms that use the brain’s plasticity to modulate the brain activity related to specific BCI tasks that use external stimuli. This approach focuses more on the subject. Specifically, to target the brain activity related to motor imagery, two stimulation methods have been applied most frequently: vibrotactile and electrical brain stimulation. In the vibrotactile stimulation, it is known that somatosensory stimulation modulates corticospinal excitability and the stimulation can increase motor evoked potentials (MEPs) [18] and electroencephalographic sensorimotor rhythms [19]. Previous studies have investigated vibrotactile stimulation’s effects during motor imagery or execution and found that it improved motor imagery BCI performance by enhancing the contralateral event-related desynchronization (ERD) over the electrode channels around the motor cortex [20–23]. For example, Ahn et al. designed a hybrid BCI paradigm combined with tactile selective attention by giving attention to vibrotactile stimulation on the left or right index fingertips during motor imagery [20]. Further, Yao et al. utilized vibrotactile stimulation for delivering tactile sensation to assist somatosensory attentional orientation (SAO) task training. They found that real tactile sensation-based training significantly improved SAO performance after the assisted training blocks [24]. Motivated by Yao’s study, Zhong et al. applied tactile sensation for assisting motor imagery BCI training by delivering sustained vibrotactile stimulation during motor imagery training blocks and found significant training effects on BCI performance compared to non-assisted motor imagery training [25]. In addition, Shu et al. applied tactile stimulation to the unilateral wrist and observed improved BCI performance with enhanced contralateral cortical activation [22]. Similarly, it was found that tactile stimulation along with motor movement (wrist extensions of paretic hand) achieved enhanced motor-related cortical activation in the alpha to beta bands and improved decoding accuracy in stroke patients compared to non-tactile stimulation conditions [23]. Moreover, recent studies have observed that frequency- or phase-specific stimulation in vibrotactile stimulation or transcranial magnetic stimulation (TMS) may produce stronger stimulation effects compared to continuous stimulation [21, 26]. In particular, Zhang et al. compared the stimulation effects of continuous vibrotactile stimulation and closed-loop stimulation activated during the rising/falling phase of alpha waves, and found that the alpha falling phase stimulation outperformed continuous and rising phase stimulation in motor imagery classification accuracy and cortical activation around the motor cortex [21]. In addition to sensory stimulation, the other stimulation method is electrical brain stimulation, such as transcranial direct current stimulation (tDCS) [27, 28]. For example, Baxter et al. investigated the stimulation effects of high-definition tDCS (HD-tDCS) by placing anode and cathode electrodes between the CP3/P3 electrode channels, and observed that anodal stimulation decreased the mean time to achieve right-hand imagery successfully and increased alpha and beta band power at the C3/CP3 channels after the stimulation session [27]. However, previous tDCS studies have reported mixed effects with respect to the ERD after the tDCS session. In particular, some previous studies found an increase in ERD in the hemisphere stimulated during motor imagery tasks [29–31], while one found a decrease in ERD following the tDCS session [32]. Still another study found that tDCS did not affect frequencies above 9 Hz [33].

Rather than developing new feature extraction algorithms, external stimulation sources may have greater potential to solve the BCI illiteracy problem, as the stimulation methods affect brain activity. In contrast, feature extraction algorithms may fail to identify the robust features if they do not exist in the brain signals recorded from BCI-illiterate individuals. However, some limitations in the stimulation-based approaches need to be addressed. One consideration is the lack of a comparative study of vibrotactile stimulation and tDCS, the stimulation method used most frequently to modulate brain activity during motor imagery and improve motor imagery BCI performance. The difficulty in conducting a comparative study derives from the stimulation paradigm. In general, vibrotactile stimulation is delivered while subjects are performing a motor imagery task because the stimulation enhances the cortical activation that motor imagery induces [21] and thus, concurrent stimulation effects can be investigated. However, with tDCS, the stimulation effects are investigated following the stimulation session because recording EEG during the stimulation session is not applicable, and appropriate stimulation is necessary to modulate brain activity. Thus, to compare the stimulation effects, the stimulation paradigms should be equal. Here, we conducted a study to compare the stimulation effects of vibrotactile stimulation and tDCS with the same stimulation paradigm by assessing the effects of the vibrotactile stimulation following the stimulation session.

Another consideration when investigating the stimulation effects is dividing the subjects according to BCI performance. Previous studies have found that inefficient (low performers) and efficient BCI users (high performers) exhibited different neurophysiological characteristics. One study incorporated brain network features into ERD to improve low performers’ motor imagery BCI performance [34]. They found that the subjects benefited from brain network features (functional connectivity), and suggested that low performers may engage in motor imagery differently than high performers, and thus existing features, such as ERD, cannot capture the engagement, although the brain network showed the possibility. Similarly, another study divided the subjects recruited into two groups based upon BCI performance, compared their brain networks on multiple network scales [35], and found significantly higher phase synchronization values in the right hemisphere during high performers’ motor imagery. In addition to brain network measures, a difference between low and high BCI performers’ frequency band power has been reported during the rest or pre-stimulus period, showing that subjects with higher alpha band or sensory motor rhythm (SMR) power during those periods are more likely to achieve better BCI performance [36–38]. Based upon these previous findings, it can be expected that vibrotactile stimulation or tDCS affects low and high performers differently because the two show different characteristics and may engage in the same task in different ways. In addition to the neurophysiological perspective, they may differ from the behavioral perspective. High performers who achieve controllable BCI performance before the stimulation session have a sense of control already, and their brain activity is optimal and stable in the task. Therefore, external changes are less likely to affect them. On the other hand, low performers do not have this sense of control, and their brain activity is not yet optimal or stable. Therefore, low performers may have more potential to change.

In this study, we compared vibrotactile stimulation and tDCS’s stimulation’s effects on brain activity related to motor imagery among inefficient BCI users. Through this study, we investigated which brain activity can be modulated by vibrotactile stimulation and tDCS and whether they can help improve low performers’ BCI performance.

## Methods

### Experimental procedure and data acquisition

**Fig. 1:**
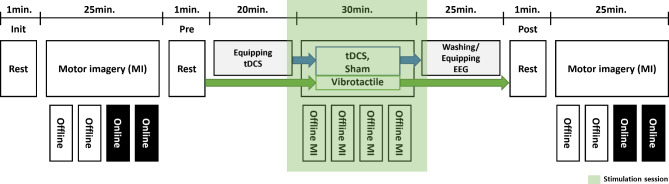
Experimental paradigm. Each participant performed the motor imagery task before and after the stimulation session

We recruited a total of 44 healthy subjects for this study, and assigned them randomly to three groups—sham (control), vibrotactile stimulation, and tDCS. This experiment was conducted in a quiet space, and the subjects were seated in a comfortable chair approximately one meter from a 24-inch screen. The subjects were asked to place both hands on a desk to prevent hand movement while they performed the motor imagery tasks. We used OpenViBE software [39] and custom-built MATLAB scripts to acquire EEG, process online signals, and present the motor imagery task. As shown in Fig. 1, EEG was recorded first for one minute during the eyes-open resting state. Thereafter, the subjects performed a left- and right-hand motor imagery task that consisted of two offline and two online blocks each. The subjects received different stimuli while performing the four offline motor imagery blocks according to their assigned groups. After the stimulation session, they performed the same motor imagery task that they performed before the stimulation session, and eyes-open resting state EEG was recorded before and after the stimulation session. EEG was recorded from 19 wired dry electrode channels, referenced by the left and right earlobes at 300 Hz (DSI-24, Wearable Sensing, USA). This experiment was approved by the Institutional Review Board at Gwangju Institute of Science and Technology (20,210,806-HR-62-03-02), and all subjects were informed about the experimental procedure and signed informed consent.

In the motor imagery task, the subjects performed left- and right-hand motor imagery rather than actual movement. The subjects were asked to imagine hand movements in a kinesthetic rather than visual way, as a previous study reported that kinesthetic motor imagery (KMI) and visual motor imagery (VMI) exhibited different brain activations [40]. Each trial began with a fixation cross for the first 1.5 s. During the next 4 s, an orange-colored circle appeared in the center of the screen, and an instruction bar appeared on the left or right side. The subjects were instructed to continue to imagine left- and right-hand movements according to the direction of the bar until the blank screen, which remained blank for 1.5 s. This trial was repeated 40 times and included shuffled left- and right-hand trials for each block. During the offline phase (two blocks), there was no motor imagery feedback. After the offline phase, the subjects’ EEG data were used to train a common spatial pattern (CSP)-based spatial filter and Fisher’s linear discriminant analysis (FLDA) classifier. Thereafter, the subjects performed the online phase, which consisted of two motor imagery blocks that showed visual feedback, for 1.5 s by moving the circle to classified directions.

For CSP-FLDA, an epoch was extracted from each trial at [1000–3500] ms to the stimulus onset, and band-pass filtered with cutoff frequencies of 8 and 30 Hz using the 4th -order Butterworth filter. However, we note that hyper-parameters for online classifiers were changed after the first ten subjects. Specifically, for the first ten subjects, CSP-FLDA was trained by 19 electrode channels and the first and last two CSP filters were selected. However, electrodes around the eyes and occipital areas often yielded notable noise with high variation attributable to non-brain activity, such as motion artifacts, eye movements, and bad contacts. As a result, for the remaining subjects, nine electrode channels around the central area, including the F3, Fz, F4, C3, Cz, C4, P3, Pz, and P4 channels, were used to train CSP, and the first and last CSP filters were selected to train FLDA. In this study, we calculated offline BCI performance to compare BCI performance with the same hyper-parameter over all subjects, as described in Sect. 2.3.

### Stimulation session design

**Fig. 2:**
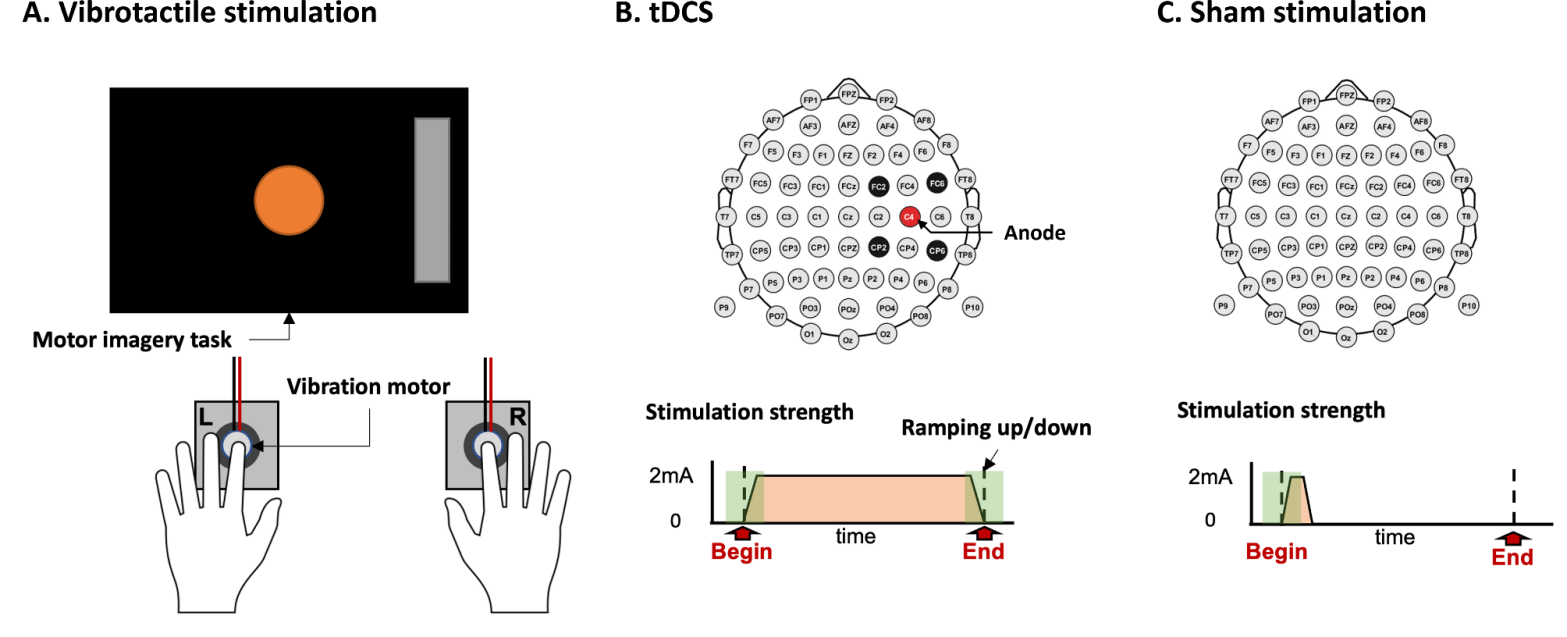
Vibrotactile stimulation, tDCS, and sham stimulation. This represents stimulation sessions for the vibrotactile stimulation, tDCS, and sham stimulation groups. (**A**) represents vibrotactile stimulation, (**B**) represents tDCS, and (**C**) represents sham stimulation

In this study, we compared the stimulation effects on brain activity modulation and relevant BCI performance. Depending upon the stimulation group, each subject performed a stimulation session in the middle of two motor imagery sessions. The stimulation groups consisted of the vibrotactile stimulation group, tDCS group, and sham stimulation group. Fig. 2 illustrates the stimulation session for each group. All stimulation group subjects performed four blocks of offline motor imagery task while receiving stimulation.

With respect to vibrotactile stimulation (Fig. 2A), two vibration motors (Model 310 − 113, Precision Microdrives, England) activated by Arduino Due board were used to deliver vibrotactile stimulation to the left and right index fingertips. Each motor is 10 mm in diameter with a 1.34G vibration amplitude. The Arduino board was triggered through serial communication with MATLAB scripts for closed loop vibrotactile stimulation. Recent studies have shown that EEG-guided stimulation can enhance the stimulation effects in vibrotactile stimulation and transcranial magnetic stimulation (TMS) by targeting motor cortex excitability states [21, 26]. Researchers have observed that stimulation triggered by the EEG alpha falling phase outperformed continuous stimulation with respect to the motor evoked potential (MEP) [26] amplitude and motor imagery BCI performance [21]. In this respect, our study applied vibrotactile stimulation according to the EEG alpha (8 ~ 13 Hz) phase at the electrode channels on the left (C3) and right motor cortex (C4) as in [21]. Specifically, during the vibrotactile stimulation session, the subjects were instructed to place their left and right index fingertips on each vibration motor. For each trial, 500ms-long epoch was extracted from the contralateral EEG channel every 50ms (with overlapping 450ms) to calculate the alpha phase. The epoch extracted was band-pass filtered in the [8–13] Hz frequency range using a 10th -order elliptical infinite impulse response (IIR) filter, and the phase was calculated using the Fast Fourier Transform (FFT)-based phase tracking algorithm, as proposed and adopted previously [21, 41]; among the FFT amplitudes, the dominant alpha frequency component between 8 and 13 Hz and the corresponding phase were used to obtain a simple sine function to predict the upcoming phase. When the phase predicted was falling, the vibration was delivered through the left or right vibration motor for 100ms according to the motor imagery class, and the inter-stimulation interval was set to 100ms. Thus, the C4 channel alpha phase was extracted for the left-hand motor imagery trials, and the left vibration motor was activated when the phase predicted was falling and the converse. With respect to the technical details, during the vibrotactile stimulation session, EEG data and event markers that represented left- or right-hand motor imagery were streamed into MATLAB scripts. To minimize delay and synchronize the EEG data and event markers, a trigger hub (Wearable Sensing, USA) was used to integrate the EEG data and event markers into the EEG device, and merged data were acquired over the wire. According to the trigger hub’s technical specification, their wired latency is less than 100µs. After the motor imagery onset, 500ms-long C3 or C4 EEG data were streamed every 50ms while calculating their phase using the FFT algorithm, which requires a simple calculation and its related delay in processing time is negligible. The vibrotactile stimulation was applied when the falling phase was detected, and it was activated using the Arduino board via serial communication within 1ms delays. However, we could not deliver the vibrotactile stimulation ideally. In practice, we delivered vibrotactile stimulation with a duration of 100ms, and the next stimulation was applied with more than 100ms inter-stimulus intervals, as set in a previous study [21]. We note that the 100ms duration was set as the minimum required stimulation duration in heuristic ways so that the subjects could perceive the stimulation and maintain attention. Consequently, the stimulation accuracy could be estimated as follows; when we assume that EEG-alpha is a 10 Hz sinusoidal signal, for each motor imagery trial, approximately 50% of the falling phases were within inter-stimulation intervals. As a previous study reported [21], we also observed that such falling phase stimulations were quite effective. However, to achieve ideal falling phase stimulation, a shorter stimulation duration should be developed while maintaining the subject’s perception, which would be worth investigating in future. After the stimulation session, the subjects performed the same motor imagery task that they did before the session.

For brain stimulation (Fig. 2B), high-definition transcranial direct current stimulation (HD-tDCS) was delivered to the motor cortex using one anode electrode and four neighbouring cathode electrodes (Starstim8, Neuroelectrics, Spain) after the EEG cap was removed. An anode electrode was placed to stimulate the contralateral motor cortex of the non-dominant hand; because all subjects in the tDCS group were right-handed, the anode electrode was placed on C4 and the cathode electrodes were placed on FC2, FC6, CP2, and CP6. To minimize pain attributable to tDCS, sufficient gel was injected, and the impedance level was maintained below 2 K${\Omega }$ throughout the stimulation session. The stimulation reached a target intensity of 2mA during a 30-second ramping period. The stimulation session continued until the subjects performed four blocks of the offline motor imagery task, although we did not record EEG during the tDCS session because of electrical interference. The subjects were informed that they could stop the stimulation at any time if they felt severe pain. In addition, the sham stimulation group (control group) subjects performed the same task as the tDCS group subjects, but they received sham, rather than actual stimulation. For the sham group, the stimulation turned off after the ramping period, and the subjects did not know whether they belonged to the tDCS or sham stimulation group. After the stimulation session, the subjects washed their hair and were re-fitted with the EEG cap. Then, they performed the same motor imagery task that they did before the stimulation session.

### BCI performance evaluation

This study used offline BCI performance because online BCI classification parameters were not the same for all subjects. To evaluate the subjects’ BCI performance, the EEG data were band-pass filtered with 8–30 Hz using the 4th -order Butterworth filter, and any 60 Hz line noise that remained was filtered out with the band-stop filter from 58 to 62 Hz. Epochs were extracted from 500 to 3500 ms to the stimulus onset, which is the time window obtained heuristically. Except for the electrodes near the eyes, those with an amplitude greater than ± 100µV were removed, and subjects who had more than 30% of bad trials among all trials were eliminated from the analysis. Finally, we chose a region of interest (ROI), the nine electrode channels (F3, Fz, F4, C3, Cz, C4, P3, Pz, and P4), for motor imagery BCI classification using visual inspection. To evaluate BCI performance, the first two motor imagery blocks (offline phase) were used for training, and the remaining two blocks were used for testing. The Riemannian minimum distance metric (MDM) [16] was used to extract features and evaluate BCI performance.

In this study, the subjects performed the motor imagery task before and after the stimulation session to evaluate stimulation effects on brain activity during motor imagery. Moreover, we divided each stimulation group into low- and high-performing groups according to their pre-stimulation BCI performance, as previous studies have observed that the two showed different neurophysiological characteristics [9, 36]. Further, our previous study suggested that low and high BCI performers should be treated differently because low BCI performers’ features decreased the ability to generalize the cross-subject BCI model significantly, while subject selection may have increased cross-subject BCI performance [42]. In addition, we assumed that low BCI performers may have greater potential to change their brain activity through learning or external stimulation, for either better or worse, compared to high BCI performers because high BCI performers’ brain activity may be stable and optimal already. To divide the subjects into low and high BCI performance groups, a previous study used the median value of the BCI performance [35], as the median value can divide groups with the same size and allow analysis to be performed on sub-groups of the same size. However, the distribution and sample size affect the median value. Accordingly, if the BCI performance data collected are biased toward high or low performance, the divided groups do not represent true high or low performance groups. Instead, we used the statistical random probability introduced in [43] and used in our previous study [42] to divide low and high BCI performers, as it can produce a random threshold based upon the number of trials and the statistical significance. With 40 test trials and α = 0.05, we obtained 60.69% as a threshold to divide low and high BCI performers. As a result, we divided each stimulation group’s subjects into low and high BCI performers based upon their pre-stimulation performance and investigated the stimulation effects in the subgroups.

### Pre-stimulus band power for motor imagery

The pre-stimulus band power was calculated from the online phase of the motor imagery task before and after the stimulation session. For clarity, we note that the pre-stimulus band power refers to the band power calculated from the window preceding the motor imagery onset in every trial, and it is distinct from the stimulation session. Thus, pre-stimulus band powers were calculated during the motor imagery task before/after the stimulation. Maeder et al. investigated the relation between the pre-stimulus sensory motor rhythm (SMR) band power and motor imagery BCI performance and observed that higher pre-stimulus SMR trials yielded significantly better performance compared to lower trials [37]. Accordingly, we compared the pre-stimulus band power before and after the stimulation session for each group to investigate the stimulus effects on the pre-stimulus band power. The online motor imagery task phases before and after the stimulation session were used to calculate and compare the pre-stimulus band power. To perform pre-processing, the epochs were extracted first from the EEG data from − 1000 to 4000 ms to the stimulus onset, band-pass filtered with 1–40 Hz, and any epochs larger than ± 100µV were removed, except for the electrodes near the eyes, and the same bad subject criterion (> 30% bad trials) was applied as in the BCI performance evaluation.

After extracting the epochs and eliminating the bad trials, we calculated the pre-stimulus band powers by the logarithmic scale of the band powers in alpha (8-13 Hz), low-beta (13-20 Hz), and high-beta (20-30 Hz) frequency bands for 1000ms preceding the stimulus onset. As the previous study selected an electrode channel from the left and right hemisphere and averaged over those electrode channels [37], we obtained the average pre-stimulus band power averaged over the C3 (left hemisphere) and C4 (right hemisphere) electrode channels. In addition, we obtained pre-stimulus band powers around the nine electrode channels (F3, Fz, F4, C3, Cz, C4, P3, Pz, and P4) used to evaluate motor imagery BCI performance. To evaluate statistical significance, pre-stimulus band powers before and after the stimulation session were compared using a paired Student’s *t*-test, and the Bonferroni correction was applied for the *p*-values obtained from the nine electrode channels.

### Functional connectivity during motor imagery

The functional connectivity between different brain regions in sensor (electrode) space can be assessed using phase synchronization. Functional connectivity allows the way the cortical regions communicate with each other and the way the information is transmitted between different regions during a cognitive task to be understood [35, 44]. One way to measure phase synchronization is by assessing phase-locking, which denotes a phase difference between two signals that remains constant for a certain period [44]. In this study, a phase locking value (PLV) between the EEG channel was calculated with the following equation introduced in [45]:

$$PL{V}_{t}=\frac{1}{N}\left|\sum _{n=1}^{N}{e}^{j\theta (t,n)}\right|$$

**Fig. 3:**
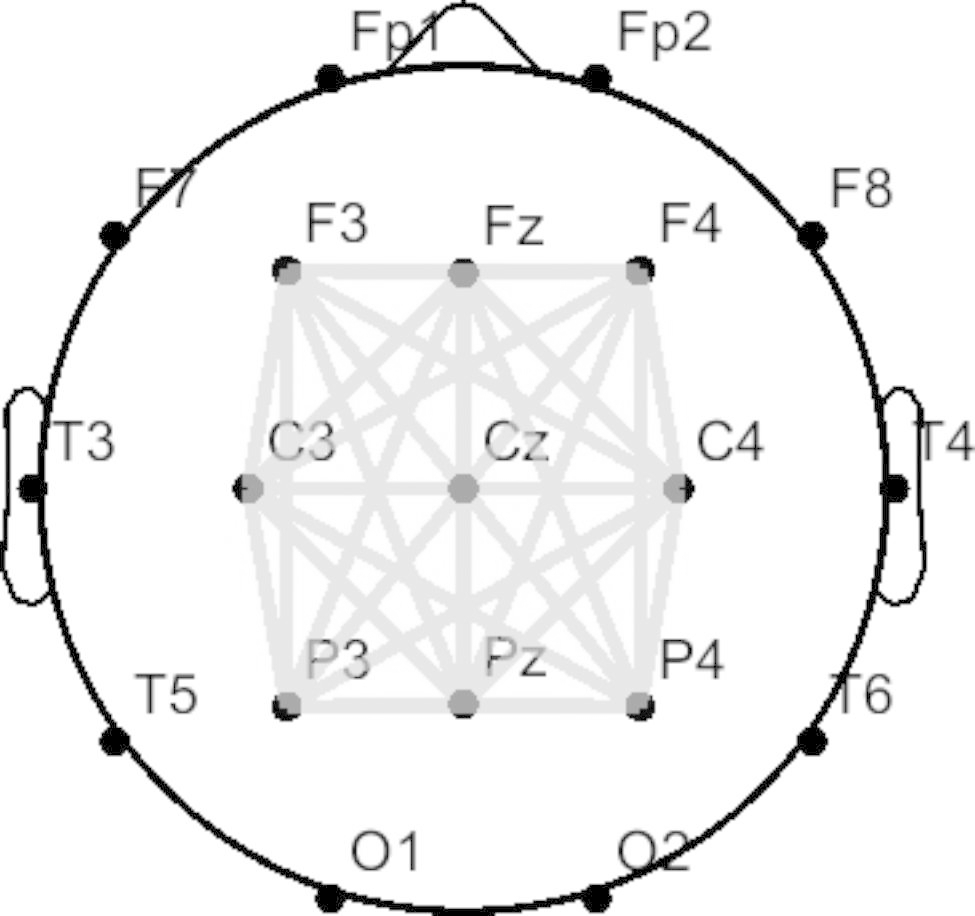
The scale of functional connectivity used in this study. This figure represents the global phase-locking value (PLV) used in this study. The global PLV was obtained by averaging all PLVs between the nine electrode channels, as illustrated by the bold lines

In which *t* stands for an extracted epoch (second) for each trial *n* up to *N*, *N* is the total number of trials, and the exponential term indicates the phase difference between two signals in the same trial, which denotes the difference between two phases extracted from the two signals [44, 45]. As a result, the PLV can measure the variability of the phase difference across trials; when the phase difference is small, the PLV is close to 1, and is near 0 otherwise. In this study, the online motor imagery task phases before and after the stimulation session were used to calculate and compare the PLV. Specifically, the epochs were extracted from the EEG data from as long as 500–3500 ms to the stimulus onset, band-pass filtered with 8–30 Hz, and the same trial rejection and subject rejection criteria were applied as in the BCI performance evaluation. With respect to scales of connectivity, this study employed a global PLV that can be obtained by averaging the connectivity over all electrodes around the central area (Fig. 3), as investigated in [46]. The nine electrode channels (F3, Fz, F4, C3, Cz, C4, P3, Pz, and P4) used to evaluate BCI performance and the pre-stimulus band power were selected. In addition, we investigated the PLV with broader frequency bands, including alpha (8-13 Hz), low-beta (13-20 Hz), and high-beta (20-30 Hz) bands by calculating the PLV within these frequency bands [35]. The PLV was calculated by MATLAB scripts using the FieldTrip toolbox [47].

The PLVs were compared in this study before and after the stimulation session using a paired Student’s *t*-test for subjects with low and high BCI performance in the sham, vibrotactile stimulation, and tDCS groups to assess the stimulation effects on the average strength of the connection around the central areas during motor imagery.

## Results

Among the 44 subjects who were assigned randomly to the three stimulation groups—sham, vibrotactile stimulation, and tDCS—bad subjects (> 30% bad trials), including one who failed to concentrate on the post-stimulation motor imagery task because of a significant delay in setup after the stimulation session, were removed from the analysis. As a result, 39 subjects remained: 12, 13, and 14 subjects in the sham, vibrotactile, and tDCS groups, respectively. Moreover, we divided each stimulation group into low and high BCI performance groups using statistical random probability. With respect to the low and high BCI performance groups, the subjects in each group who achieved a performance lower than 60.69% were assigned to the low BCI performance group, and the remaining subjects were assigned to the high BCI performance group. As a result, sham group subjects were divided into 6 low performers and 6 high performers, vibrotactile stimulation group subjects were divided into 10 low performers and 3 high performers, while the tDCS group subjects were divided into 10 low performers and 4 high performers. Although we assigned the stimulation groups randomly, the vibrotactile stimulation and tDCS groups included more low performers than the sham stimulation group.

### Stimulation effects on BCI performance

**Fig. 4:**
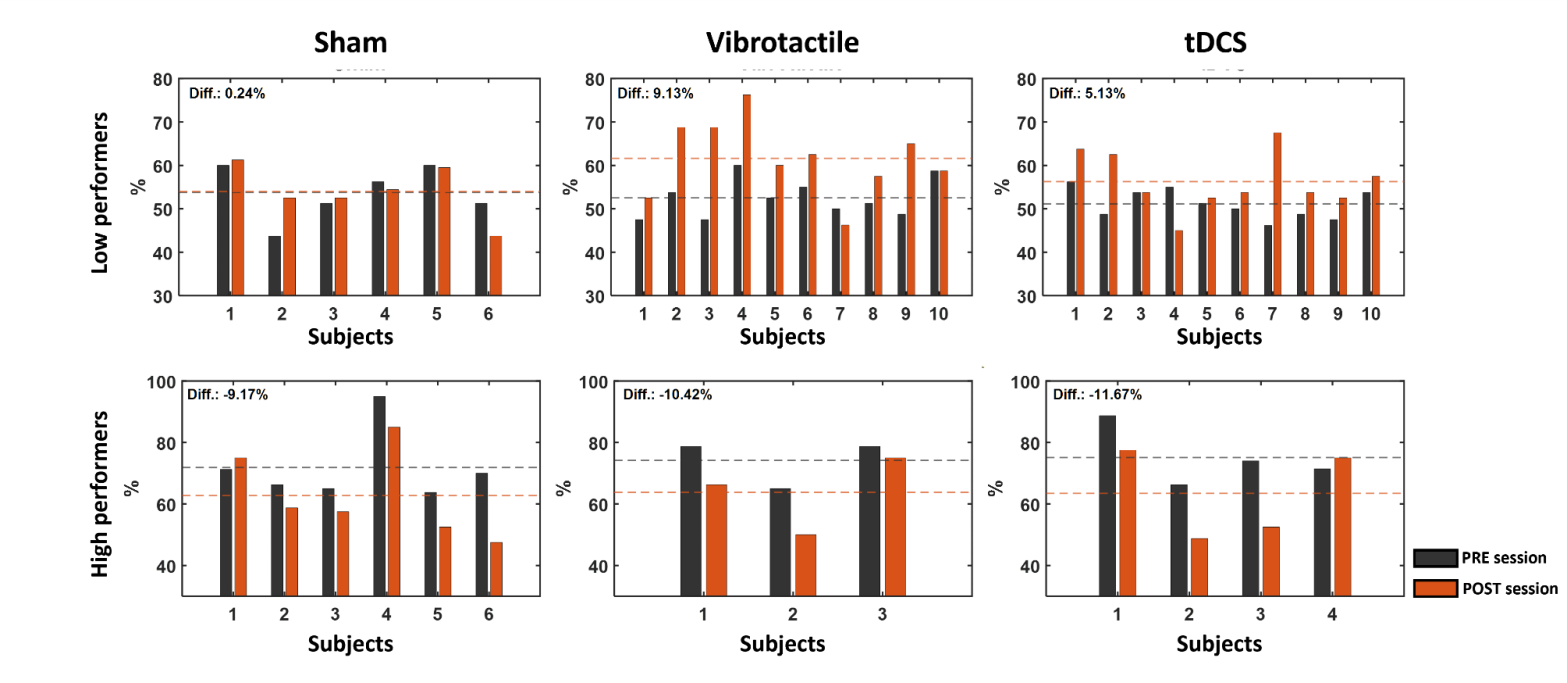
Motor imagery BCI performance before and after the stimulation. This figure represents BCI performance changes after the stimulation session for each stimulation and performance group. Black bars represent pre-stimulation motor imagery BCI performance, orange bars represent post-stimulation performance, and dashed lines represent the averages of pre- and post-stimulation BCI performance

Motor imagery BCI performances during pre-stimulation and post-stimulation are depicted in Table 1; Fig. 4. Low performers in the sham stimulation group achieved 53.75% (43.75 ~ 60%), and high performers achieved 71.88% (63.75 ~ 95%) before the pre-stimulation session. After the sham stimulation session, low performers achieved 53.99% (43.75 ~ 61.25%), showing no significant change. However, the high performers’ BCI performance decreased to 62.71% (47.5 ~ 85%). On the other hand, the low performers in the actual stimulation groups, the vibrotactile stimulation and the tDCS groups, showed improved performance. For the vibrotactile stimulation group, low performers achieved 52.5% (47.5 ~ 60%), and high performers achieved 74.17% (65 ~ 78.75%). After the vibrotactile stimulation session, the low performers’ BCI performance increased to 61.63% (46.25 ~ 76.25%), while the high performers’ BCI performance decreased to 63.75% (50 ~ 75%). Finally, for the tDCS group, low performers achieved 51.12% (46.15 ~ 56.25%), and high performers achieved 75.11% (66.25 ~ 88.75%). After the tDCS session, the low performers’ BCI performance increased to 56.25% (46.25 ~ 76.25%), and the high performers’ BCI performance decreased to 63.44% (48.75 ~ 77.5%).

**Table 1:** Motor imagery BCI performance

		Sham stimulation		
	LOW		HIGH	
	PRE	POST	PRE	POST
**BCI performance (%)**	53.75 (43.75 ~ 60%)	53.99 (43.75 ~ 61.25%)	71.88 (63.75 ~ 95%)	62.71 (47.5 ~ 85%)
		**Vibrotactile stimulation**		
	**LOW**		**HIGH**	
	**PRE**	**POST**	**PRE**	**POST**
**BCI performance (%)**	52.5 (47.5 ~ 60%)	61.63 (46.25 ~ 76.25%)	74.17 (65 ~ 78.75%)	63.75 (50 ~ 75%)
		**tDCS**		
	**LOW**		**HIGH**	
	**PRE**	**POST**	**PRE**	**POST**
**BCI performance (%)**	51.12 (46.15 ~ 56.25%)	56.25 (45 ~ 67.5%)	75.11 (66.25 ~ 88.75%)	63.44 (48.75 ~ 77.5%)

We compared BCI performance before and after the stimulation session using a paired Student’s *t*-test over all stimulation groups. The results showed that the low performers in the vibrotactile stimulation group achieved significantly improved BCI performance after the stimulation session (*p* = 0.0053 < 0.01). The low performers in the tDCS group achieved improved BCI performance as well, but the difference was not significant. The high performers’ BCI performance decreased over all stimulation groups, and the sham stimulation group showed a significant decrease (*p* = 0.048 < 0.05).

### Stimulation effects on pre-stimulus band power

**Fig. 5:**
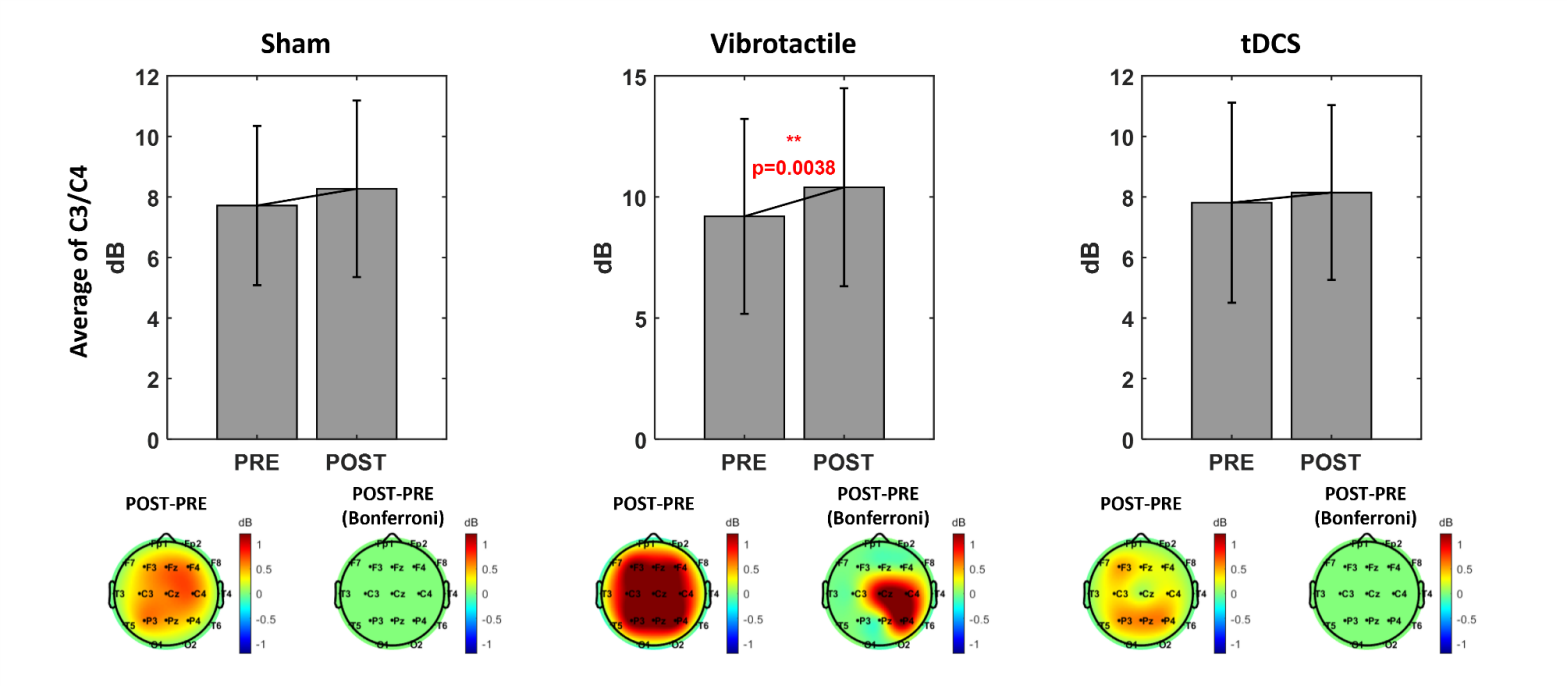
Pre-stimulus alpha band power before and after the stimulation. The figure shows the pre-stimulus alpha band power changes after the stimulation session for the low performers in each stimulation group. Each scalp topography displays only nine selected electrode channels, and the remainder are zero-padded. The left scalp topography represents the difference in the pre-stimulus alpha band power, and the right scalp topography displays only the significant electrode channels

Among the pre-stimulus band powers, including the alpha (8-13 Hz), low-beta (13-20 Hz), and high-beta (20-30 Hz) bands, only the pre-stimulus alpha band power was statistically significant. In this respect, Fig. 5 represents the pre-stimulus alpha band power changes after the stimulation session for low performers in each group. For the sham stimulation group, the mean of the C3 and C4 pre-stimulus alpha band powers increased from 7.72 ± 2.43dB to 8.27 ± 2.77dB after the sham stimulation session, but the increase was not statistically significant. In the scalp topographic view, the pre-stimulus alpha band powers increased after the sham stimulation session, but no electrode channel showed a statistically significant difference. For the tDCS group, the average pre-stimulus alpha band power increased from 7.81 ± 8.15dB to 8.15 ± 3.09dB after the tDCS session, but this difference was not statistically significant either. As observed in the sham stimulation group, the scalp topography shows an alpha increment, but again, it was not statistically significant. The vibrotactile stimulation group showed the same alpha increment as observed in the other groups and remained statistically significant; the average pre-stimulus alpha band power increased from 9.20 ± 3.81dB to 10.40 ± 4.05dB after the vibrotactile stimulation session (*p* = 0.0038). The scalp topography also showed the alpha increment, and a paired Student’s *t*-test with the Bonferroni correction revealed significant differences in the Cz, C4, and P4 electrode channels.

The high performers in each group demonstrated no significant changes in the pre-stimulus alpha band power over all stimulation groups. In the sham stimulation group, the average alpha band power increased from 10.24 ± 3.09dB to 11.02 ± 2.98dB after the sham stimulation session, and the average alpha band powers increased from 11.72 ± 1.03dB to 12.03 ± 0.45dB after the stimulation session in the vibrotactile stimulation group and from 10.53 ± 3.77dB to 10.89 ± 3.92dB in the tDCS group. However, there was no significant change in the pre-stimulus band power, except for the significant decrement in the high-beta (13-20 Hz) band power in the vibrotactile stimulation group (*p* = 0.0469). Further, there was no significant change in the scalp topography over all stimulation groups. These results are consistent with the finding in BCI performance that only low performers in the vibrotactile stimulation group achieved significantly improved BCI performance after the stimulation session.

### Stimulation effects on functional connectivity

**Fig. 6:**
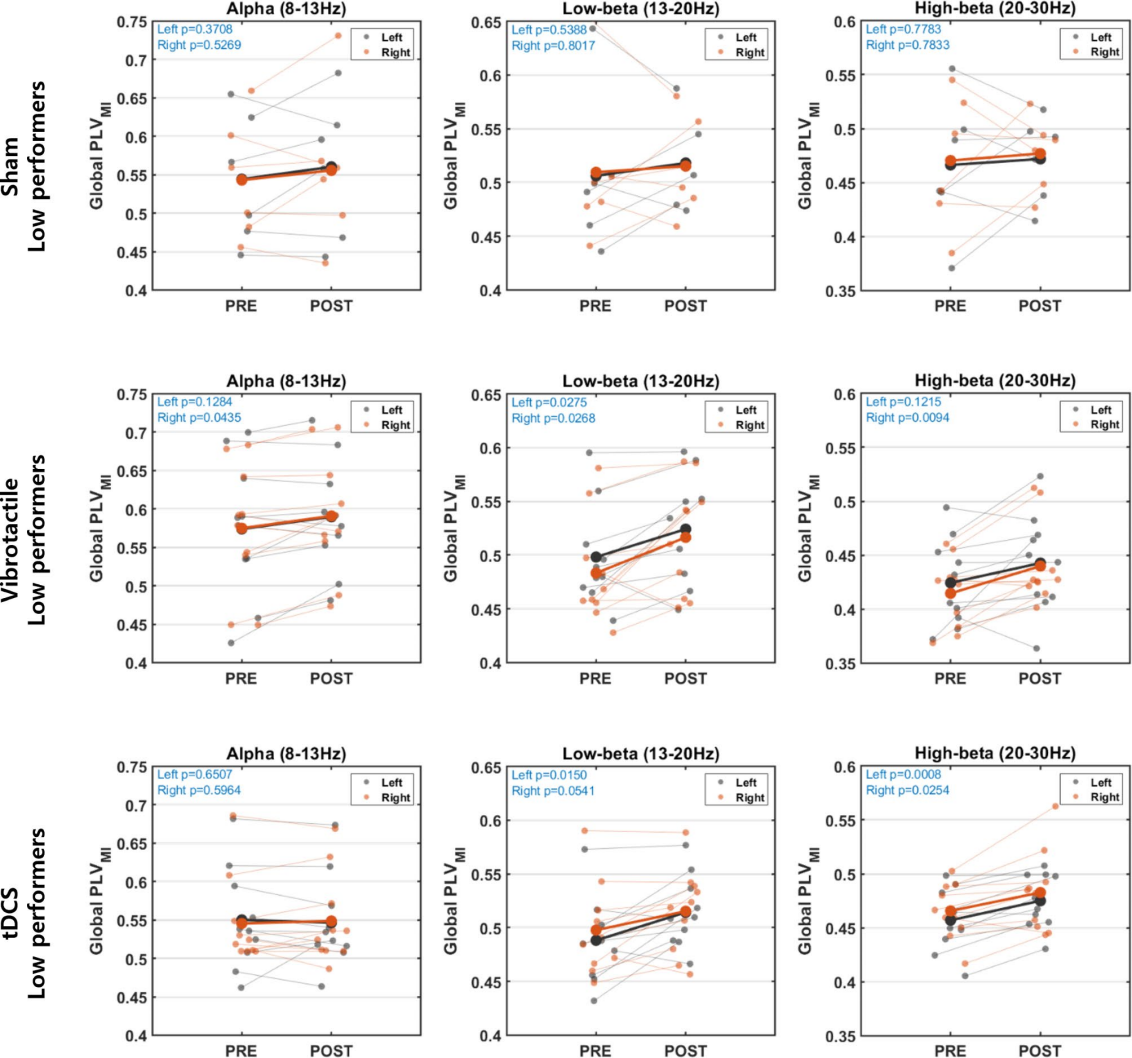
Global PLVs before and after the stimulation. The figure represents the global PLVs averaged over the connections between the nine electrode channels’ (F3, Fz, F4, C3, Cz, C4, P3, Pz, and P4) changes after the stimulation session for low performers in each stimulation group

We investigated the stimulation effects on brain activity through functional connectivity assessed by global PLVs during motor imagery before and after the stimulation session around sensorimotor areas, including the nine electrode channels (F3, Fz, F4, C3, Cz, C4, P3, Pz, and P4) in various frequency bands, the alpha (8-13 Hz), low-beta (13-20 Hz), and high-beta (20-30 Hz) bands. Fig. 6 represents the global PLV changes for low performers in each stimulation group during left- and right-hand motor imagery. In addition, the global PLVs before and after the stimulation session were compared using a paired Student’s *t*-test.

The average global PLVs over the motor imagery classes and frequency bands for the low performers showed no significant changes after the sham stimulation session, and yielded no consistent trends, as the global PLVs increased for some subjects and decreased for others. However, the vibrotactile stimulation and the tDCS groups showed statistically significant differences. Specifically, in the vibrotactile stimulation group, the global PLV within the alpha (8-13 Hz) band increased significantly during right-hand imagery after the vibrotactile stimulation session (*p* = 0.0435), while the increment in the global PLV during left-hand imagery was not significant. Within the low-beta (13-20 Hz) band, the global PLVs increased significantly for both left- (*p* = 0.0275) and right-hand imagery (*p* = 0.0268). The high-beta (20-30 Hz) also showed a significant increment in the global PLV during right-hand imagery (*p* = 0.0094). In the tDCS group, the global PLV within the alpha band did not change significantly, while significant increments were observed in the low-beta and high-beta bands. Within the low-beta band, the global PLV during left-hand imagery increased significantly (*p* = 0.015), and it increased during right-hand imagery as well, but not significantly. Within the high-beta band, the global PLVs increased significantly for both left- and right-hand imagery after the tDCS session (*p* = 0.0008 and *p* = 0.0254, respectively).

Except for the sham group, the high performers demonstrated no statistically significant change over all stimulation groups and frequency bands. In this group, the global PLV in the low-beta band during left-hand imagery increased significantly after the sham stimulation session (*p* = 0.0203). However, there was no significant change in the global PLVs in the vibrotactile stimulation and tDCS groups. Still, we note that the numbers of low and high performers were not balanced because this study divided the subjects using statistical random probability, which is the constant value rather than the median value. As a result, there were three and four high performers in the vibrotactile stimulation and tDCS group, respectively, so the statistical tests for the high performers may not be reliable. However, this study’s focus was to investigate the stimulation effects for low BCI performers with respect to BCI performance and brain activity. Therefore, we set a threshold with a constant value to divide the low and high BCI performers rather than dividing them equally.

In the functional connectivity analysis, we observed a consistent trend in the results of BCI performance and pre-stimulus band power by dividing subjects into low and high BCI performers. With respect to motor imagery BCI performance, only low performers in the vibrotactile stimulation group showed significant improvement. The tDCS group also demonstrated improved BCI performance, although it was not statistically significant. The low performers in the sham stimulation group showed no change after the sham stimulation. Similarly, a significant increase in the pre-stimulus alpha band power was observed in the low performers only in the vibrotactile stimulation group. Eventually, the global PLVs over the sensorimotor areas showed significant increases for low performers in the vibrotactile stimulation and tDCS groups. In contrast, no significant change was observed in the sham stimulation group. High performers’ BCI performance decreased across all stimulation groups, and no significant change was observed in the brain activity except in a small number of cases.

## Discussion

This study compared vibrotactile stimulation and tDCS’s stimulation effects on BCI performance and brain activity. In addition, subjects in each stimulation group were divided into low and high BCI performers under the assumption that the external stimulation would affect the two differently. Our results demonstrated the stimulation effects according to stimulation types, and BCI efficiency with respect to BCI performance and brain activity.

### Stimulation effects

We investigated stimulation effects with respect to motor imagery BCI performance and brain activity, including the pre-stimulus band power and global functional connectivity. First, with respect to BCI performance, the performance of low performers in the vibrotactile stimulation and tDCS groups improved by as much as 9.13% for the vibrotactile and 5.13% for the tDCS groups, respectively, after the stimulation session, but only the vibrotactile stimulation group subjects showed statistically significant differences (*p* = 0.0053). During the stimulation session, all groups of subjects performed four blocks of offline motor imagery tasks while they received sham, vibrotactile stimulation, and tDCS. As a result, all subjects performed four blocks of motor imagery before, during, and after the stimulation session, and this repetition could affect performance changes as the subjects became accustomed to the task. Therefore, the results should be investigated further to determine whether the performance changes were attributable to the repetition of motor imagery alone rather than vibrotactile stimulation and tDCS. In this respect, the sham stimulation group subjects performed the same task, in that they also performed motor imagery tasks during the sham stimulation session. In contrast to the vibrotactile stimulation and tDCS groups, the sham stimulation group subjects showed no notable change in BCI performance, but their BCI performance improved by as much as 0.24% after the sham stimulation session. Therefore, we can infer that the repetition of the motor imagery task without external stimulation did not affect the changes in low performers’ BCI performance. Although BCI performance needs to be improved further, the vibrotactile stimulation group subjects achieved performance significantly better than random chance, 60.69% obtained by the current test trial numbers, and statistically significant at α = 0.05, after the stimulation session, indicating that longitudinal stimulation may improve BCI performance.

High performers suffered large performance decrements after the stimulation session in all groups, although it may not be relevant because there were only three and four high performers in the vibrotactile stimulation and tDCS groups, respectively. In contrast to low performers, high performers had achieved controllable BCI performance before the stimulation session already, indicating that they can generate distinct brain activity during motor imagery, so they had a good strategy and sense of control. External stimulation may be unnecessary for these individuals, or the fatigue attributable to the task’s repetition may have affected them more than the stimulation. Another assumption is that a longer stimulation session may be necessary to modulate brain activity, as a single day session may be too short.

With respect to the pre-stimulus band power, Maeder et al. found that trials with a higher SMR amplitude during the pre-stimulation period yielded better BCI performance than lower amplitude trials [37]. They suggested that ongoing SMR may play a key role in motor imagery BCI and other motor-related tasks in general. Inspired by their findings, we assumed that external stimulation could enhance the pre-stimulus band power and improve BCI performance simultaneously. In this study, we investigated the pre-stimulus (1000ms preceding the stimulus onset) band power, including alpha (8-13 Hz), low-beta (13-20 Hz), and high-beta (20-30 Hz) bands, before and after the stimulation session. We observed increased pre-stimulus alpha band power after the stimulation session across all groups of subjects, and it can be expected that cognitive and physical fatigue attributable to the long experimental tasks increased the alpha band power overall. However, statistical tests revealed a significant alpha increase only in low performers in the vibrotactile stimulation group (*p* = 0.00038). This is consistent with the fact that low performers’ BCI performance improved significantly in the vibrotactile stimulation group. Moreover, as Maeder et al. observed, high performers showed higher pre-stimulus alpha band power than low performers. As a result, pre-stimulus alpha plays a key role in motor imagery, and modulating the pre-stimulus alpha may help improve motor imagery BCI performance. No significant change was observed in the low- and high-beta bands, except that high performers’ pre-stimulus high-beta band power decreased in the vibrotactile stimulation group (*p* = 0.0469).

In addition to pre-stimulation band power, this study was inspired by previous studies that have investigated the relation between BCI performance and functional connectivity [34, 35] in an effort to determine EEG characteristics other than ERD that play a role in motor imagery, as previous studies have reported tDCS’s mixed effects on ERD [29, 32, 33]. It is unclear whether ERD is a key feature for both low and high performers, or whether they engage in a motor imagery task differently and generate different brain activity. However, it is true that event-related desynchronization/synchronization (ERD/ERS) is an index of motor imagery used widely [4, 30]. We calculated contralateral ERD/ERS from motor imagery EEG (C3 ERD/ERS during right-hand imagery and C4 ERD/ERS during left-hand imagery) for each subgroup in the same manner used in motor imagery BCI classification. As expected, high performers showed clear ERD during motor imagery in the 8-30 Hz frequency range before the stimulation session, while only a few low performers exhibited ERD. However, we could not find a clear pattern attributable to ERD/ERS changes after the stimulation session consistent with the BCI performance increase/decrease, and no significant changes were observed in ERD/ERS averaged over subjects. Further, we observed some subjects who achieved decreased BCI performance after the stimulation session, although clear ERD was still observed in the 8-30 Hz frequency range in high performers. Conversely, we observed some subjects who achieved improved BCI performance after the stimulation session, even though there was no notable difference in ERD/ERS after the session in low performers. Based upon these results, it may be expected that performance modulation was not sufficient to elicit ERD/ERS changes. Some low performer’s performance modulations were still within the range of low BCI performance, and high performers’ performance modulation was still within the range of high BCI performance, although it is not clear how low or high BCI performance could elicit the corresponding ERD/ERS changes. Moreover, contralateral ERD/ERS could be observed in different brain areas (channels), frequencies, and time ranges because of inter-subject variability, which makes it difficult to generalize the results of this analysis. In summary, these results demonstrated that low performers who achieved improved BCI performance showed increased global connectivity and pre-stimulus alpha band powers, while ERD/ERS changes did not show the same pattern. As discussed earlier, this may indicate that low and high performers engage in a motor imagery task differently, which induces different EEG features during the task. To achieve a more precise analysis, in-depth investigation should be conducted that focuses more on ERD/ERS with a large number of subjects, which we will investigate in our future work.

With respect to the brain activity during motor imagery other than ERD, Zhang et al. found that brain network measures could improve low performers’ BCI performance, suggesting that only these features may capture their engagement in motor imagery [34]. Leewis et al. investigated the difference between low and high performers by assessing the average strength of all connections on different scales of the brain network [46, 48, 49], including the global, large, and local scales, rather than complex graph theory measures. Although our study did not consider all network scales because of a lack of available electrode channels, this study investigated the global PLVs during motor imagery calculated by averaging all connections within the electrode set (F3, Fz, F4, C3, Cz, C4, P3, Pz, and P4) that have a high signal-to-noise ratio (SNR). We observed that with low performers, vibrotactile stimulation and tDCS increased global PLVs after the stimulation session. With vibrotactile stimulation, low performers exhibited significantly increased global PLVs in alpha (*p* = 0.0435), low-beta (*p* = 0.0268), and high-beta (*p* = 0.0094) in right-hand imagery, and low-beta (*p* = 0.0275) in left-hand imagery. The low performers in the tDCS group demonstrated significantly increased global PLVs in both left- (*p* = 0.015 for low-beta and *p* = 0.0008 for high-beta) and right-hand (*p* = 0.0254 for high-beta) imagery over the low- and high-beta bands. On the other hand, the sham stimulation group subjects showed no significant change in global PLVs in any frequency bands and motor imagery classes. Instead, high performers showed a significant increase in left-hand imagery at low-beta (*p* = 0.0203), while those in the vibrotactile stimulation and tDCS groups showed no significant changes. As a result, there were clear differences in functional connectivity, as observed in BCI performance and pre-stimulus band power, according to stimulation types and BCI efficiency (low and high performers).

The results of BCI performance, pre-stimulus band power, and functional connectivity showed the same trend, in that the low performers in the vibrotactile stimulation and tDCS groups showed significant improvement, while low performers in the sham stimulation group showed no significant changes, although the pre-stimulus band power and functional connectivity differed significantly in the different frequency bands. Moreover, high performers demonstrated different effects compared to low performers in BCI performance, pre-stimulus band power, and functional connectivity following the stimulation session, indicating that dividing subjects into low and high performers was appropriate to investigate the stimulation effects.

### Stimulation paradigm

We designed the same stimulation paradigm for vibrotactile stimulation and tDCS in this study. BCI performance and brain activity in both groups were investigated before and following the stimulation session rather than investigating the stimulation effects concurrently. A hybrid technique can be more effective in vibrotactile stimulation because it can enhance ERD when combined with motor imagery [20, 21]. However, in practice, a hybrid method requires the subjects to receive multiple stimulation types each time they perform BCI tasks, which can tire the users more despite the fact that incorporating vibrotactile stimulation may improve their performance. Instead, if stimulation effects persist after the stimulation session, and the subjects achieve the BCI performance desired after multiple stimulation sessions over multiple days or months, they may be able to perform the BCI task more effectively without the stimulation. Although this study included only a single day session, and thus, the stimulation effects were investigated immediately after the stimulation session, our results demonstrated that vibrotactile stimulation could modulate brain activity and improve BCI performance.

### Limitations and future directions

One limitation in this study is that the sample size was too small to investigate low and high performers in depth. The study was designed with a lengthy timeline to evaluate stimulation effects in a single day session. High performers may have become bored and lost attention during the lengthy experiment because they had achieved controllable BCI performance already, while low performers may have maintained their motivation longer because they want to achieve improved BCI performance. In addition, as we discussed, the external stimulation may affect low and high performers differently because they may engage in a motor imagery task and generate different brain activity, as in [34, 35]. However, we note that although we recruited a large number of subjects (N = 44) and 39 remained for the analysis, the subjects were divided into groups according to the stimulation types, sham, vibrotactile, and tDCS. Moreover, each group was sub-divided according to their initial BCI performance to investigate low and high performers separately. As a result, there were only three and four high performers, respectively, in the vibrotactile stimulation and tDCS groups, so it is necessary to observe the trend found in high performers carefully. Therefore, one can argue that dividing subjects may be not appropriate. However, as we observed, the sub-divided subjects showed different characteristics with respect to BCI performance, pre-stimulus band power, and functional connectivity. In addition, those features’ stimulation effects were distinguished for low and high performers. Previous studies have found that low and high performers showed different characteristics with respect to band powers [9, 37] and functional connectivity [35]. Further, we observed that low and high performers exhibited different effects depending upon vibrotactile stimulation or tDCS. However, these results should be interpreted with caution because the number of high performers was insufficient, and performance was compared during only a single day session. For a more precise interpretation, high and low performers should be recruited equally in a sufficient number, and the experimental paradigm should be shortened by conducting multiple day sessions in future work.

Moreover, a single day session may not be sufficient for some subjects to entrain their brain activity. This analysis showed significantly improved performance in the vibrotactile stimulation group. In contrast, the tDCS group did not show a statistically significant change although the subjects achieved improved performance, and there were significant changes in their brain activity. However, we cannot conclude that vibrotactile stimulation is superior to tDCS based upon this result, because the stimulation time required to modulate brain activity may differ, and the tDCS group subjects spent extra time preparing for the tDCS session. Therefore, multiple sessions over longer periods may be necessary to investigate the stimulation effects precisely.

In terms of vibrotactile stimulation, we delivered 100 ms vibrations for the falling phases of alpha waves. Even though the stimulation is initiated at the falling phase, it may be hard to target the falling phase of alpha wave accurately without affecting other phases, which is another limitation of this study. Actually, this stimulation duration was set practically as the minimum required duration for subjects so that they could perceive and maintain attention to the stimuli. We note that a previous study delivered 20 ms vibrotactile stimulation for every falling phase of alpha waves, which theoretically could stimulate the falling phase only [21]. They used voice coil tactor that is controlled by PC soundcard and an audio amplifier, while this study used vibration motors controlled by Arduino Due board via voltage and electrical current. Thus, as future work, it is compelling to conduct more experiments for various stimulation durations (less than 100 ms), with targeting the falling phase of alpha wave. For such study, elaborate tactors, such as voice coil tactor used in [21], may be utilized for accurate targeting.

With respect to functional connectivity analysis, we calculated the average strength of PLVs over the nine electrode channels around the sensory motor areas (F3, Fz, F3, C3, Cz, C4, P3, Pz, and P4), as the EEG cap used in this study had a sparse electrode montage and thus, there were no fronto-central or centro-parietal electrodes. Therefore, we calculated a very simple index to investigate the stimulation effects rather than using complex connectivity measures, such as those from graph theory. Using a greater number of electrode channels can provide more freedom in a functional connectivity analysis when investigating network features and scales, such as inter/intra-hemisphere analysis.

## Conclusion

We conducted a comparative study here to compare different stimulation modalities’ effects on inefficient BCI users’ motor imagery BCI performance and brain activity. Our results showed that the vibrotactile stimulation and tDCS groups achieved improved BCI performance after the stimulation session, but the improvement in the tDCS group was not statistically significant. Consistently, functional connectivity and pre-stimulus alpha band power increased significantly in the vibrotactile stimulation group. Moreover, we found that efficient and inefficient BCI users exhibited different stimulation effects, in that most efficient users demonstrated decreased BCI performance after the stimulation. These findings indicated that inefficient and efficient BCI users experience different stimulation effects as well as differ in BCI efficiency, although this should be investigated with more subjects over a longer period. In addition, this study could contribute to research designed to improve BCI performance by modulating brain activity, rather than developing a new feature extraction algorithm, by providing changes in various brain activities during motor imagery and a detailed analysis, such as subgroup analyses according to stimulation types and BCI efficiency. At the same time, this study raised research questions, such as how much performance modulation in motor imagery BCI must be achieved to elicit similar ERD/ERS modulations, which will be investigated in future work. We believe such studies will contribute to solving the BCI-illiteracy problems by allowing inefficient BCI users to achieve controllable performance, in addition to achieving near-perfect BCI performance for users who have achieved controllable performance already.

## Data Availability

The datasets and/or analysed during the current study are not publicly available because additional analysis in different perspectives is undergoing. However, it may be available from the corresponding author after request review.
